# Does prophylactic sotalol and magnesium decrease the incidence of atrial fibrillation following coronary artery bypass surgery: a propensity-matched analysis

**DOI:** 10.1186/1749-8090-1-6

**Published:** 2006-03-03

**Authors:** V Aerra, M Kuduvalli, AN Moloto, AK Srinivasan, AD Grayson, BM Fabri, AY Oo

**Affiliations:** 1 Department of Cardiothoracic Surgery. The Cardiothoracic Centre Liverpool, UK; 2 Department of Research and Development. The Cardiothoracic Centre Liverpool, UK; 3 Senior Clinical Information Analyst, Clinical Governance Department. The Cardiothoracic Centre-Liverpool, Thomas Drive, Liverpool, L14 3PE, UK

## Abstract

**Background:**

Atrial fibrillation can occur in up to 40% of patients undergoing coronary surgery.

**Methods:**

We retrospectively analysed 103 consecutive coronary surgery patients under the care of one surgeon between April 2003 and September 2003. These patients received 40 mg of sotalol orally twice daily from the first post-operative day for 6 weeks and 2 g of magnesium intravenously immediately post surgery and on the first post-operative day. We developed a propensity score for the probability of receiving sotalol and magnesium after coronary surgery. 89 patients from the sotalol and magnesium group were successfully matched with 89 unique coronary surgery patients who did not receive either sotalol or magnesium with an identical propensity score.

**Results:**

Preoperative characteristics were well matched between groups. There was no significant difference with respect to in-hospital mortality between groups (sotalol and magnesium 1.1% versus control 4.5%; p = 0.17). The incidence of atrial fibrillation in the sotalol and magnesium group was 13.5% compared to 27.0% in the controls (p = 0.025).

**Conclusion:**

The combination of sotalol and magnesium can significantly reduce the incidence of post-operative atrial fibrillation following coronary surgery.

## Introduction

Atrial Fibrillation is the most common complication following cardiac surgery with a reported incidence between 20% and 40% [1,2]. It usually occurs between the second and fourth post-operative day. However, although this complication occurs frequently, the mechanism behind its development is less understood.

Numerous studies have identified and enumerated a variety of risk factors for the development of atrial fibrillation. These include increased age, male gender, history of atrial fibrillation, discontinuation of preoperative βeta-blocker therapy, congestive heart failure, electrolyte depletion (low potassium and magnesium), cardiopulmonary bypass, left atrial dysfunction, severity of coronary artery disease, respiratory disease, and pulmonary vein venting [1-7].

In an effort to prevent the occurrence of atrial fibrillation in the post-operative period, various treatment modalities have been implemented indicating its unclear pathophysiology. The key role of βeta blockers in prevention of atrial fibrillation is well recognised in many randomised control trials [8,9]. Sotalol has an acceptable safety profile and is emerging as a key drug in the prevention of this complication [9,10]. The role of magnesium supplements is less clear [11-17].

The present study was done to examine the beneficial role of sotalol and magnesium prophylaxis in the prevention of atrial fibrillation in routine coronary artery bypass graft (CABG) surgery.

## Materials and methods

### Patient population

Between 1^st ^April 2003 and 30^th ^September 2003, 103 consecutive patients undergoing first time isolated CABG surgery under the care of one surgeon (AYO) were routinely administered sotalol and magnesium (*see sotalol and magnesium*). These patients were matched to a control group taken from the remaining 487 consecutive patients who received first time isolated CABG surgery performed during the same time period by other surgeons at our institution (*see statistical methods*).

### Exclusions

Patients undergoing CABG that was in addition to heart valve repair or replacement, resection of a ventricular aneurysm or other surgical procedure were not included. Also excluded were patients who had received previous cardiac surgery or patients with a history of atrial arrhythmias.

### Data collection

Definitions and data collection methods are available from . Data was collected prospectively during the patient's admission as part of routine clinical practice and entered into our cardiac surgery registry on the variables listed in Table 1. Post-operative atrial fibrillation, in-hospital mortality, and length of hospital stay were also documented.

**Table 1:** Propensity-matched patient and disease characteristics

	SM (n = 89)	Control (n = 89)	P Value
Age (years)	65.9 (59.2 – 70.1)	66.4 (59.2 – 71.9)	0.58
Female (%)	28.1	25.8	0.74
Body mass index (kg/m2)	27.1 (24.9 – 30.7)	27.1 (24.7 – 30.2)	0.81
Ejection fraction <30%	5.6	4.5	0.73
Triple-vessel disease (%)	75.3	74.2	0.86
Left main stem disease (%)	23.6	24.7	0.86
Angina class IV (%)	19.1	14.6	0.42
Diabetes (%)	23.6	29.2	0.39
Respiratory disease (%)	37.1	33.7	0.64
Renal dysfunction (%)	6.7	5.6	0.76
Peripheral vascular disease (%)	9.0	7.9	0.79
Current smoker (%)	20.2	20.2	>0.99
Previous myocardial infarction (%)	46.1	51.7	0.45
Hypercholesterolaemia (%)	95.5	95.5	>0.99
Hypertension (%)	57.3	55.1	0.76
Emergent procedure (%)	1.1	1.1	>0.99
Preoperative β-blockers (%)	66.3	64.0	0.75
Preoperative diuretics (%)	33.7	36.0	075
Preoperative heart rate (bpm)	69.1 (61.8 – 81.9)	70.3 (60.6 – 83.2)	0.57
Additive EuroSCORE	4 (2 – 5)	3 (2 – 5)	0.63
Number of grafts (n/patient)	3 (2 – 4)	3 (3 – 4)	0.61
Right-CAD (%)	76.4	71.9	0.49
LIMA (%)	95.5	96.6	0.69
CPB not used (%)	77.5	69.7	0.23

SM, Sotalol and Magnesium; CPB, Cardiopulmonary Bypass; EuroSCORE, European System for Cardiac Operative Risk Evaluation; CAD, Coronary Artery Disease; LIMA, Left Internal Mammary Artery. Categorical variables are shown as a percentage. Continuous variables are shown as a median with 25th and 75th percentiles.

### Sotalol and Magnesium

Patients received 40 mg of sotalol orally twice daily from the first post-operative day for 6 weeks and 2 g of magnesium intravenously immediately post-coronary surgery and on the first post-operative day. No patients from the control group received sotalol and/or magnesium.

### Outcome definitions

Post-operative atrial fibrillation was defined as the occurrence of new atrial fibrillation in the absence of pre-operative persistent or paroxysmal atrial fibrillation prior to hospital discharge. In-hospital mortality was defined as death within the same hospital admission regardless of cause.

### Statistical methods

In order to assess the effect of sotalol and magnesium, we performed a propensity-matched cohort analysis [18]. To do this, logistic regression was used to develop a propensity score for sotalol and magnesium group membership. The propensity score included the following variables: age, sex, body mass index, respiratory disease, renal dysfunction, peripheral vascular disease, diabetes, severity of angina and dysponea, hypertension, hypercholesterolaemia, smoking status, extent of disease, ejection fraction, priority and use of cardiopulmonary bypass. The C statistic, which is equivalent to the receiver-operating characteristic curve, for the propensity model was 0.85. Sotalol and magnesium patients were matched with control patients who had an identical 5-digit propensity score. If this could not be done, we then proceeded to a 4-, 3-, 2-, or 1-digit match [19].

Continuous variables are shown as median with 25^th ^and 75^th ^percentiles and comparisons were made with Wilcoxon rank-sum tests. Categorical variables are shown as a percentage and comparisons were made with Chi-square tests. A forward stepwise logistic regression analysis was performed to identify risk factors for atrial fibrillation, which was adjusted for the propensity score. In all cases a P value < 0.05 was considered significant. All statistical analysis was performed retrospectively with SAS for Windows Version 8.2 (SAS Institute, Cary, NC).

## Results

Of the 590 patients who underwent first time isolated CABG at our institution during the study period, 28.0% developed post-operative atrial fibrillation. Of the 103 patients who received sotalol and magnesium, 13.6% developed atrial fibrillation compared to 31.0% in the other patients (p < 0.001).

We were able to successfully match 89 patients who received sotalol and magnesium to unique control patients who did not. Of these, 5 (5.6%) were 5-digit matches, 11 (12.4%) were 4-digit matches, 40 (44.9%) were 3-digit matches, 24 (27.0%) were 2-digit matches, and 9 (10.1%) were a 1-digit match. The patient and disease characteristics of the propensity-matched patients are shown in Table 1.

There was no significant difference with respect to in-hospital mortality between groups (sotalol and magnesium 1.1% versus control 4.5%; p = 0.17). The median length of hospital stay in the sotalol and magnesium group and control group were both 7 days [(25^th ^and 75^th ^percentiles: 6 to 9); p = 0.38].

**Figure 1:**
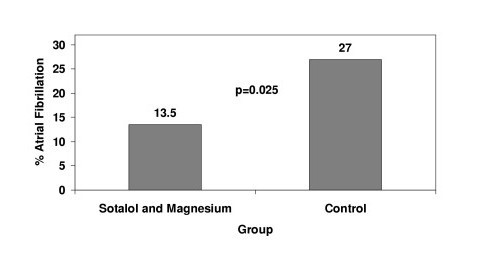
Propensity-matched incidence of atrial fibrillation.

**Figure 2:**
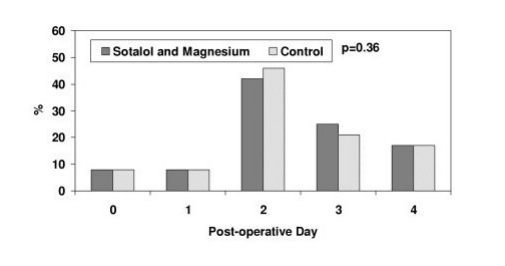
Time of occurrence of post-operative atrial fibrillation in propensity-matched patients.

The incidence of atrial fibrillation in the sotalol and magnesium group was significantly lower compared to the control group (Figure 1; p = 0.025); with an odds ratio of 0.42 (95% confidence interval: 0.19 to 0.91). The time of occurrence of atrial fibrillation was similar between the propensity-matched patients (Figure 2; p = 0.36).

A logistic regression analysis confirmed the beneficial effect of sotalol and magnesium for reducing post-operative atrial fibrillation (Table 2).

**Table 2:** Independent risk factors for post-operative atrial fibrillation

	Odds Ratio †	95% Confidence Interval	p Value
Female	0.17	0.05 – 0.61	0.006
SM group	0.39	0.18 – 0.89	0.025
Body mass index #	0.89	0.81 – 0.99	0.025

SM, Sotalol and Magnesium

† Adjusted for the propensity score

# for each additional unit

## Comment

Numerous studies have identified and enumerated a variety of pre-, intra- and post-operative risk factors [1-7], with the aim of being able to change practice and reduce the occurrence of atrial fibrillation. Several treatment modalities have been investigated and have shown the effectiveness of either prophylactic amiodarone or sotalol in reducing this complication [9,10]. A meta-analysis, by Wurdeman, concluded that compared to placebo, amiodarone and sotalol were both effective in reducing post-operative atrial fibrillation when used prohpylactically, and that both were equally as effective as each other [9]. Sotalol has non-selective beta-blocking as well as class III antiarrhythmic activity. In addition, it has no interaction with the other beta blockers and so patients on pre-operative beta blockers other than Sotalol could be easily switched to Sotalol for atrial fibrillation prophylaxis. Sotalol is associated with an increase in QTc interval but reported incidence of torsades de pointes is very low. It can sometimes induce profound bradycardia and hypotension and should be avoided in low cardiac output states.

Recently, Forlani and associates [20] found that the incidence of atrial fibrillation after coronary operations was significantly reduced by the administration of combination sotalol and magnesium therapy. They showed in a randomised control trial of 207 consecutive CABG patients, that administration of sotalol alone and magnesium alone significantly reduced the incidence of atrial fibrillation, but more importantly, that when combined together, the incidence of atrial fibrillation was reduced to 1.9%.

The presence of hypomagnesemia following cardiac surgery is well described and has been shown to result in higher atrial myocardium excitability and an increased incidence of post-operative atrial fibrillation [14]. The role of magnesium supplementation in reducing atrial fibrillation has been extensively studied with varying results.

Hazelrigg and colleagues [11] concluded that prophylactic magnesium supplementation does not significantly reduce atrial arrhythmias, and that the only benefit occurred on the first post-operative day. Sinilarly, Parikka [12] concluded that magnesium was not sufficient for decreasing the incidence of atrial fibrillation. In a recent randomised, double-blinded, placebo controlled trial, Geertman found that magnesium in addition to sotalol, was not more effective than sotalol alone in the prevention of tachyarrhythmias after CABG. They also found that serious bradyarrhythmias were observed in the magnesium-treated group of patients [13].

However, other reports in the literature have highlighted the potential role of magnesium for reducing arrhythmias post cardiac surgery [14-17]. Toraman and colleagues [15] found that patients who received magnesium had a reported incidence of atrial fibrillation of just 2% compared to 21% in the control group. They point out that several studies that failed to show the benefit of magnesium in reducing atrial fibrillation were not well randomised with patients in the magnesium group being older and having more preoperative palpitations and arrhythmias [12]. Alghamdi [16] published a systematic review and meta-analysis in 2005 which concluded that intravenous magnesium was associated with a significant reduction in the incidence of atrial fibrillation after coronary artery bypass surgery, with a relative risk of 0.64 (95% CI 0.47 to 0.87; p = 0.004). Another meta-analysis published in the same year by Miller and colleagues showed a similar result with patients receiving magnesium having an atrial fibrillation rate of 18% compared to 28% in the control group (p < 0.001) [28].

Our own study confirms that the combination of sotalol and magnesium can significantly reduce the incidence of post-operative atrial fibrillation, with an incidence of 13.5% compared to 27% in a propensity-matched cohort. But, unlike the study by Forlani [20], which had strict exclusion criteria, this study is based on routine clinical practice.

There are some limitations which need to be considered when drawing conclusions from this report. The first is that it is an observational study and therefore could have some degree of selection bias. The retrospective nature of the study cannot account for unknown variables affecting the outcomes that are not correlated strongly with the variables used in the propensity matching. Although, propensity score adjustment is no substitute for a properly designed randomised control trial, retrospective comparisons with propensity score adjustment are more versatile and may be more widely applicable to routine clinical practice [18]. Perhaps one of the most important limitations is inextricable confounding caused by the sotalol and magnesium patients being performed completely by one surgeon. Even after careful application of multivariate analyses and propensity-matching, it remains difficult to distinguish between surgeon and treatment differences. However these results are highly suggestive of a role for the use of sotalol and magnesium in CABG and we are already embarking on a randomised control trial to confirm or refute the findings of this observational study.

In conclusion, the combination of sotalol and magnesium can significantly reduce the incidence of post-operative atrial fibrillation following coronary artery bypass surgery.
